# Explainable 3D vision transformer framework for detecting brain anomalies in neuropsychiatric subtypes of Parkinson's disease

**DOI:** 10.3389/fninf.2026.1810223

**Published:** 2026-07-15

**Authors:** Carlos Jiménez-Farfán, Alisson Constantine-Macias, Isaac León, Juan Pisco-Jordán, Luis Ramos-Pozo, Enrique Pelaez, Ana Tapia-Rosero, Edwin Valarezo-Añazco, Ignacio Obeso, Ana Briones-Chonillo, Maria José Miranda, Luis Yepez-Guerra, Francis R. Loayza

**Affiliations:** 1 Facultad de Ingeniería en Electricidad y Computación (FIEC), Escuela Superior Politécnica del Litoral, ESPOL, Campus Gustavo Galindo, Guayaquil, Ecuador; 2 Cognitive Control & Habit Lab, Cajal Neuroscience Centre—CSIC, Alcalá de Henares, Madrid, Spain; 3 Hospital Guayaquil Abel Gilbert Pontón, Guayaquil, Ecuador; 4 Hospital General del Norte de Guayaquil Los Ceibos, Instituto Ecuatoriano de Seguridad Social (IESS), Avenida del Bombero, Guayaquil, Ecuador; 5 Laboratory of Neuroimaging and Bioengineering, FIMCP, Escuela Superior Politécnica del Litoral, ESPOL, Campus Gustavo Galindo, Guayaquil, Ecuador

**Keywords:** apathy, explainable AI, impulse control disorders, neuropsychiatric PD, Parkinson's disease

## Abstract

**Introduction:**

Neuropsychiatric manifestations, such as impulse control disorders (ICD) and apathy, are common in Parkinson's disease (PD) and significantly impact patient quality of life, yet their underlying neuroanatomical substrates remain poorly understood. This study presents an explainable artificial intelligence (XAI) framework to investigate these phenotypes using structural MRI.

**Methods:**

We employed a 3D Vision Transformer (MobileViT-3D) trained on T1-weighted images from 364 drug-naive PD patients, stratified into four subgroups: idiopathic PD (PD), PD with apathy (PD + A), PD with ICD (PD + ICD), and PD with both conditions (PD + A + ICD). To interpret the model's decision making, Guided Backpropagation and Integrated Gradients were applied to generate saliency maps, followed by a multi-level statistical analysis. Based on these patterns, volumes of interest (VOI) were defined. Conventional morphometric (e.g., cortical thickness) and radiomic texture features were obtained from each VOI applied to each subject.

**Results:**

Voxel-wise analysis of saliency maps suggested group-level differences after correction for multiple comparisons (p ≤ 0.05 FWE) in prefrontal, temporal, and cerebellar regions of the neuropsychiatric conditions compared to PD. While no significant group differences were observed for morphometric features, radiomic texture analysis identified eight features associated with differences between the PD + A + ICD group and idiopathic PD. These features included texture metrics indicative of increased heterogeneity and complexity.

**Discussion:**

Overall, these findings provide preliminary evidence that a combined XAI and radiomic approach may help identify subtle, texture-based neuroanatomical signatures associated with the co-occurrence of apathy and ICD in early PD that are not readily captured by conventional univariate analyzes. This framework may support the generation of clinically relevant hypotheses regarding the neural correlates of heterogeneous neuropsychiatric symptoms in PD.

## Graphical Abstract

**Figure:**
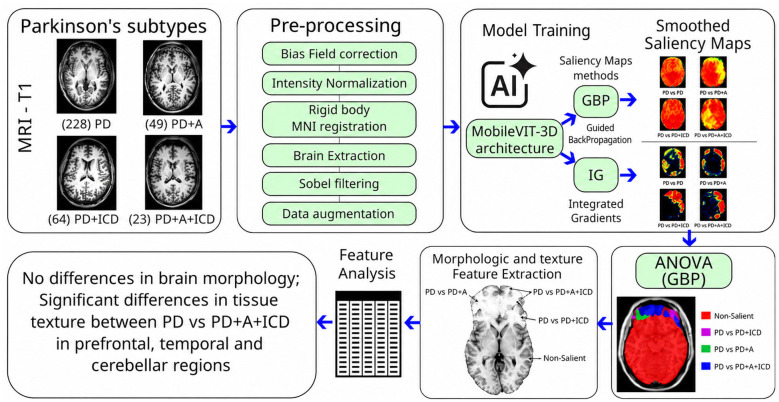

## 1 Introduction

Neuropsychiatric manifestations, such as impulse control disorders (ICD) and apathy, are prevalent in both the general population (Grant et al., 2005; Odlaug and Grant, 2010) and Parkinson's disease (PD) patients (Vriend et al., 2014; Martini et al., 2018). ICDs are characterized by the inability to suppress urges or behaviors despite adverse consequences, while apathy is defined as a lack of motivation and goal-directed activities. These symptoms substantially impair the quality of life in PD patients and exacerbate disease management challenges. Although ICDs and apathy represent distinct clinical phenotypes, emerging evidence suggests they frequently co-occur in PD (Scott et al., 2020), potentially sharing overlapping neuroanatomical substrates (Maggi et al., 2024).

Despite these different phenotypes, apathy and ICD have been associated with dopamine replacement therapy in PD (Starkstein, 2012), along with other factors such as male sex, depression, history of substance abuse, younger age at the time of the onset of the disease, and severe motor symptoms (Pedersen et al., 2009; Voon et al., 2006; Weintraub et al., 2010; Voon et al., 2011). Anatomical studies suggest that these manifestations involve widespread cortical deterioration beyond the basal ganglia, particularly affecting the frontal cortex (Reijnders et al., 2010; Maggi et al., 2024). For example, Reijnders et al. (2010) identified a reduction in gray matter density in regions such as the pre-SMA, inferior parietal gyrus, inferior frontal gyrus, and insula in PD patients with high apathy scores. Similarly, Maggi et al. (2024) reported distinct gray matter reductions for the neuropsychiatric PD subgroups in prefrontal and medial brain regions.

Despite these findings, current studies face limitations in systematically analyzing and interpreting the neural patterns associated with these neuropsychiatric symptoms. Recent advances in artificial intelligence (AI), particularly transformer-based architectures, have shown promise for neuronal Volume of Interest (VOI) imaging analysis. Transformer models are capable of capturing long-range spatial dependencies within complex anatomical structures, addressing the limitations of traditional Convolutional Neural Networks (CNNs), which rely on localized receptive fields. In particular, vision transformer-based models such as TransUNet (Chen et al., 2024), UNETR (Hatamizadeh et al., 2022b), and Swin-UNETR (Hatamizadeh et al., 2022a) have set new performance benchmarks in the segmentation of volumetric MRI data. Furthermore, transformer architectures like MedViTV2 (Nejati Manzari et al., 2026) and DeiT (Touvron et al., 2021) utilize self-attention mechanisms to capture long-range spatial dependencies across the entire brain for enhancing diagnostic transparency, while achieving state-of-the-art performance in classification.

However, while these architectures manage to achieve a great diagnostic performance, these architectures often operate as “black-box” systems, which poses a significant challenge for clinical adoption, particularly in safety critical domains such as healthcare. Explainability is essential to address these challenges and ensure that AI models are interpretable and trustworthy for medical professionals. Recent techniques have emerged for AI explainability, providing information on which regions of the image the model focuses on during classification tasks. For CNNs, widely used methods include Guided Backpropagation (Springenberg et al., 2014), Integrated Gradients (Sundararajan et al., 2017), Guided Integrated Gradients (Gu et al., 2021), and SmoothGrad (Smilkov et al., 2017). These methods primarily compute the gradients of the output concerning the feature maps in the last convolutional layer, highlighting the areas that contribute to the model's predictions.

In contrast, explainability techniques for transformer-based architectures often leverage the attention mechanisms inherent to the model. Methods such as Grad-CAM (Selvaraju et al., 2017) and Guided Grad-CAM++ (Chattopadhay et al., 2018) have been adapted to transformer models to identify regions of importance by analyzing gradient based activations of token representations within self attention layers (Chefer et al., 2021). These saliency maps provide valuable information about which parts of the input image are most relevant to the decision making process. Although these techniques have shown promise in tasks that involve natural images, limited efforts have focused on explaining medical images, particularly brain imaging data. Such methods are essential for uncovering neuroanatomical patterns underlying model decision making in neuroimaging.

According to previous studies (Maggi et al., 2024; Reijnders et al., 2010), neuroanatomical patterns associated with ICD and apathy in PD exhibit specific regional differences in gray matter density. These findings suggest a potential role for explainability tools in identifying clinically meaningful spatial patterns related to overlapping neuropsychiatric phenotypes in PD. However, most applications of XAI in DL frameworks for PD analysis, focus on generalized disease diagnosis or motor progression, as summarized in Table 1. For example, Camacho et al. (2023) and Hussain et al. (2025) applied XAI techniques on 3D DL models to identify relevant brain regions, but their analyzes focused on binary classification between PD patients and HC. As a result, the use of XAI to investigate overlapping neuropsychiatric phenotypes in PD remains limited. Luna et al. (2022) partially addressed this gap by employing Grad-CAM to visualize salient patterns in the orbitofrontal cortex associated with ICD. While their results highlight the potential of explainability methods for identifying clinically relevant brain regions in neuropsychiatric phenotypes in PD, they still fall short of providing comprehensive and fully interpretable neuroanatomical insights.

**Table 1:** Recent works on explainability methods on DL for PD localization using T1-Weighted MRI.

References	Dataset	Methodology	Research gap
Basaia et al. (2025)	PPMI	Implemented a 3D CNN with Grad-CAM to locate progression biomarkers in 3D T1-weighted MRIs.	Focus on motor severity and progression of the disease instead of neuropsychiatric phenotypes in PD.
Hussain et al. (2025)	PPMI	Proposed a hybrid deep learning architecture combining a Swin Transformer and CNN to classify Parkinson's disease from MRI images, using Grad-CAM to highlight brain regions contributing to the model's predictions.	Focus on explanation of local PD vs HC classification rather than investigating neuroanatomical patterns associated with neuropsychiatric manifestations such as ICD or apathy.
Camacho et al. (2023)	Used 13 datasets, including: PPMI, OASIS3, BioCog, COMPASS-ND, and others.	Trained a 3D CNN and applied SmoothGrad to identify influential brain regions on 3D T1-weighted MRIs.	Focus on binary PD vs HC classification, without analyzing overlapping neuropsychiatric phenotypes in PD.
Luna et al. (2022)	PPMI	Utilized an ensemble of CNNs with Grad-CAM to localize patterns in PD vs PD + ICD patients in T1-weighted MRI slices.	The explainability analysis focused in MRI slices instead of 3D volumetric data. Apathy was not analyzed.

Building upon these advancements, this work proposes a methodological framework designed to enhance explainability in deep learning models applied to neuropsychiatric manifestations in PD. To improve model interpretability and ensure attribution consistency, we integrate two complementary gradient-based attribution techniques, Guided Backpropagation and Integrated Gradients, with statistical analyzes, feature extraction, and machine learning tools, enabling a robust, data-driven characterization of neuroanatomical patterns.

The goal of this paper is to generate anatomically coherent and clinically interpretable explanations that support hypothesis generation regarding the neuroanatomical substrates related to impulsivity and apathy in PD. This approach provides anatomically grounded insights into the neural patterns associated with ICD and apathy in PD, bridging the gap between AI-based neuroimaging and clinical understanding.

## 2 Materials and methods

### 2.1 The dataset

Previous work (Maggi et al., 2024) reported a neuropsychiatric follow-up assessment using the Parkinson's Progression Markers Initiative (PPMI) database. PPMI is a publicly accessible research dataset, subject to registration and data use agreement. The PPMI study was approved by the institutional review boards of all participating sites, and all participants provided written informed consent. PD patients were grouped according to the presence of apathy (PD + A), impulse control disorders (PD + ICD), the co-occurrence of both symptoms (PD + A + ICD), and idiopathic PD without manifestations of neuropsychiatric disorders. Following the methodology established in our previous work (Maggi et al., 2024), the clinical labels were derived from assessment instruments at the baseline visit using a binary classification rule. Specifically, apathy was identified by a score of ≥1 on item 1.4 of the MDS-UPDRS Part I, while ICDs were determined by a score of ≥1 on the Questionnaire for Impulsive-Compulsive Disorders in Parkinson's Disease (QUIP). The PD + A + ICD subgroup was defined by the concurrent presence of both conditions, requiring patients to meet the threshold for both instruments simultaneously. All clinical assessments and T1-weighted MRI acquisitions were concurrent, ensuring that the patients were drug-naive at the time of evaluation. A total of 364 MRI T1-weighted structural images were collected from the PPMI database using each patient's ID and group. The images were downloaded exclusively from untreated patients in all cases of neuropsychiatric disorders. MRI acquisition followed the standardized PPMI structural T1-weighted protocol implemented across participating sites, as defined by the PPMI Imaging Core. Detailed acquisition procedures and sequence parameters are provided in the official PPMI MRI Procedure Manual and MRI Technical Operations Manual Parkinson's Progression Markers Initiative (PPMI) Imaging Core (2022, 2025).

In summary, the composition and baseline demographic and clinical characteristics of the dataset are presented in Table 2. The varying group sizes reflect the inherent variability in the prevalence of these neuropsychiatric manifestations within the PPMI cohort. While the subgroups demonstrated strong comparability across age, sex, education, disease duration, and global cognitive status.

**Table 2:** Baseline demographic and clinical characteristics of the study cohort.

Variable	PD (A)	PD + A (B)	PD + ICD (C)	PD + A + ICD (D)	Statistic	*p*-value
	(*n* = 228)	(*n* = 49)	(*n* = 64)	(*n* = 23)	(χ^2^)	
Demographics						
Age (years)	61.45 ± 9.51	60.98 ± 9.81	60.23 ± 10.45	60.65 ± 10.27	0.608	0.894
Biological sex (M/F)	149 / 79	34 / 15	40 / 24	15 / 8	0.394	0.941
Education (years)	15.70 ± 3.05	15.06 ± 2.82	15.37 ± 2.75	15.30 ± 2.90	1.966	0.580
Clinical variables						
Disease duration (m)	25.96 ± 22.00	23.18 ± 21.28	24.09 ± 21.18	30.67 ± 30.00	0.424	0.935
H&Y stage	1.56 ± 0.51	1.67 ± 0.48	1.43 ± 0.50	1.78 ± 0.42	11.018	0.012*
UPDRS-III Score	20.95 ± 9.31	22.90 ± 7.83	18.92 ± 8.00	21.48 ± 6.31	7.657	0.054
Cognitive status						
MoCA score	27.22 ± 2.34	27.04 ± 2.08	26.69 ± 2.49	27.35 ± 1.90	3.395	0.335

Data are presented as mean ± standard deviation unless otherwise stated. Bold *p*-values indicate statistical significance (*p* < 0.05).

^*^Post-hoc analysis revealed significant differences for H&Y Stage between group D and group C (D > C).

PD, Parkinson's disease; A, apathy; ICD, impulse control disorders; H&Y, Hoehn and Yahr; UPDRS, unified Parkinson's disease rating scale; MoCA, Montreal cognitive assessment; m, months.

### 2.2 Data preprocessing

A Python environment was used for image pre-processing of the neuroimaging data used in subsequent analyzes. The Ants library (Avants et al., 2009) was employed for the pre-processing procedure, which consisted of the following: first, the conversion from DICOM to NIfTI format. Next, MRI intensity gradients were minimized using a bias field correction method to reduce field inhomogeneities across different scanner manufacturers. Following this, intensity normalization was applied to standardize the contrast across diverse acquisition protocols. To prevent brain deformation and preserve local texture relationships, the images were co-registered with the Montreal Neurological Institute (MNI) template using rigid body registration with six degrees of freedom (DoF). This approach ensures that anatomical proportions and voxel-wise intensity distributions are maintained without the distortions often introduced by non-linear warping (Carré et al., 2020).

A mask was used to extract the brain from all images, minimizing potential biases introduced by non-brain structures during subsequent analyzes. Finally, a Sobel Edge Detection (SED) filter was applied. This algorithm enhances edges by calculating the gradient of image intensity, which involves computing both the magnitude and direction of the gradient at each pixel. This process emphasizes areas where the intensity changes abruptly, such as edges and boundaries. The SED algorithm detects edges in both horizontal and vertical directions, combining this information into a single metric (Rezai-Rad and Aghababaie, 2006). The application of SED produced a more compact representation of cortical geometry, reducing image dimensionality while preserving relevant anatomical structure. Crucially, this algorithm also acts as a feature-level harmonization step, by focusing on local intensity gradients rather than absolute intensities; the resulting maps are more invariant to global scanner specific biases, varying signal-to-noise ratios or contrast differences (Carré et al., 2020; Arunnehru et al., 2025). This enhanced the visibility of brain boundaries and facilitated clearer interpretation of neuroanatomical patterns that transcend site related batch effects, providing a clearer and more interpretable basis for the subsequent explainability analyzes.

#### 2.2.1 Data augmentation

To mitigate data sparsity, we implemented a multi-pronged augmentation strategy. This encompassed geometric transformations via 18 unique rotations along sagittal plane axes and a duplication step using a denoising-based augmentation method. Concurrently, to address class imbalance, a targeted random subsampling was applied, retaining 20% of the over-represented PD samples and 75% of the PD + ICD class samples, while preserving all samples from the other minority subgroups. These combined geometric, intensity based, and sampling based procedures were designed to enhance dataset diversity and balance, thereby reducing potential sampling bias for subsequent model training.

### 2.3 Model architecture

**Figure 1:**
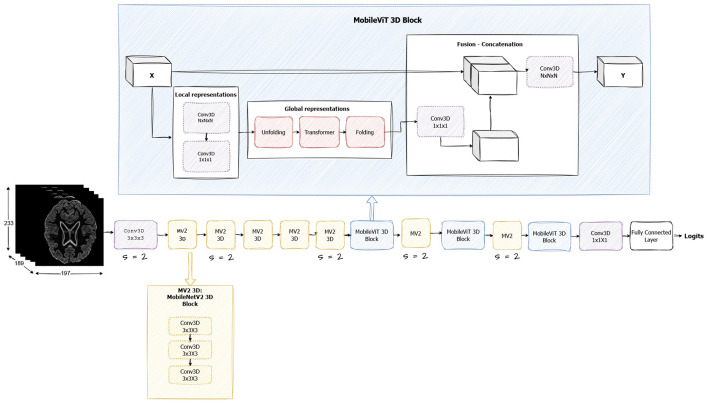
Overview of the MobileViT-3D architecture, previously introduced in our earlier work (Jiménez-Farfán et al., 2024). The model consists of three MobileViT 3D blocks, each containing a convolutional layer, a transformer layer designed to capture long-range dependencies, and a folding layer for dimensionality reduction. Additionally, a MobileNetV2 block was employed for feature extraction. The output from this block was fused with the outputs of the MobileViT 3D blocks through a fusion-concatenation layer. Finally, a global representation layer aggregates these features into a unified global embedding, which serves as the basis for the explainability analyzes conducted in this study.

The MobileVIT-3D model from a previous work (Jiménez-Farfán et al., 2024) was leveraged. This model was based on the MobileViT architecture (Mehta and Rastegari, 2022) for volumetric data by extending its convolutional and transformer blocks into a fully 3D implementation (see Figure 1). This model employs a hybrid convolution-transformer architecture by integrating 3D convolutional layers, for local feature extraction, with 3D self-attention mechanisms to capture long range global dependencies.

While a comprehensive assessment of the base architecture, training parameters, and computational cost is detailed in our foundational work (Jiménez-Farfán et al., 2024), establishing a high level of predictive reliability on our specific cohort is a strict prerequisite for a valid interpretation of the model's anatomical focus. To contextualize the explainability analyzes, we first rigorously evaluated the model's baseline classification performance. As summarized in Table 3, a 5-fold cross-validation demonstrated high stability and consistency across data splits, yielding a macro-averaged accuracy of 89.92% and an AUROC of 97.79%.

**Table 3:** Macro-averaged cross-validation performance of the 3D MobileViT model across 5 folds.

Metric	CV mean ± Std (%)
Accuracy	89.92 ± 5.89
Sensitivity	89.92 ± 5.89
Specificity	95.06 ± 2.04
AUROC	97.79 ± 1.65

Data are presented as mean ± standard deviation.

**Figure 2:**
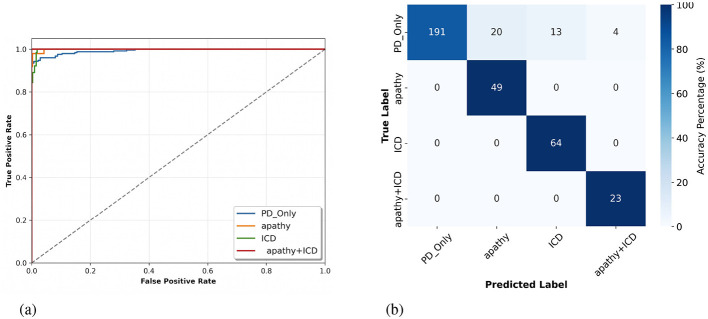
Performance evaluation of the MobileViT-3D model on the unaugmented baseline MRIs. **(a)** Multiclass ROC curves on the unaugmented baseline MRIs. **(b)** Confusion matrix on the unaugmented baseline MRIs.

Since our primary objective extended beyond pure classification to the identification of key neuroanatomical regions, we additionally evaluated the model across the entire original, unaugmented dataset. This step was taken exclusively to analyze the spatial feature attribution on the actual MRI scans, ensuring the anatomical fidelity of the explanations. Figure 2 illustrates the model's behavior in this specific context, displaying the multiclass Receiver Operating Characteristic (ROC) curves alongside the normalized confusion matrix. On this full dataset, the ensemble model demonstrated robust discriminative ability across all four categories, achieving an aggregate AUC-ROC of 0.99 and a class-wise sensitivity and specificity exceeding 83%.

As explicitly detailed in the confusion matrix and Table 4, the model achieved perfect sensitivity (100%) for the Apathy, ICD, and Apathy + ICD minority classes when evaluated on the unaugmented baseline MRIs. However, due to the relatively small size of these cohorts, these perfect classification rates are subject to small-sample variance and must be interpreted cautiously. To transparently quantify this statistical uncertainty, we computed 95% confidence intervals (CIs) for our per-class metrics utilizing the Wilson score interval. Unlike standard bootstrap resampling, which can artificially report zero variance on perfectly classified small samples, the Wilson score incorporates the sample size (*N*) to provide a mathematically rigorous lower confidence bound. For instance, it indicates that the true real-world sensitivity for the Apathy + ICD cohort (*N* = 23) may reasonably be as low as 85.7%. This statistical bounding constitutes an important contextualization of the present study, as exceptionally high quantitative performance on limited datasets may obscure underlying model biases or shortcut learning. In this context, the spatial explainability and saliency analysis presented in the following section becomes particularly relevant for examining whether the model's predictions are genuinely driven by meaningful morphological patterns.

**Table 4:** Detailed per-class predictive performance of the final ensemble model evaluated on the unaugmented baseline MRIs.

Class	Sensitivity (95% CI)	Specificity (95% CI)	Precision (%)	F1-score (%)	Support (*N*)
PD_Only	83.4 (78.3–87.5)	100.0 (97.3–100.0)	100.00	90.95	228
Apathy	100.0 (92.7–100.0)	93.4 (90.2–95.6)	69.01	81.67	49
ICD	100.0 (94.3–100.0)	95.6 (92.8–97.4)	82.05	90.14	64
Apathy + ICD	100.0 (85.7–100.0)	98.6 (96.8–99.4)	82.14	90.20	23

Sensitivity and specificity are reported with 95% confidence intervals (CI) calculated using the Wilson score method to rigorously account for small-sample variance.

Given this established level of predictive robustness, this 3D MobileViT model serves as a highly capable foundation for our XAI framework. The remainder of this study focuses on examining the internal feature representations and attention distributions of this architecture to uncover and interpret anatomical patterns biologically, underscoring the explainability of transformer-based encoding in clinical neuroimaging.

### 2.4 Explainability algorithms

We applied two complementary explainability algorithms to characterize the anatomical patterns emphasized by the network. The objective of this analysis was to identify the anatomical regions and structural features that are highly relevant to the model's differentiation between classes. The analysis sought to determine whether the explainability maps highlighted biologically meaningful patterns within cortical, subcortical, and white-matter structures. The following procedure was implemented to conduct this investigation.

#### 2.4.1 Guided backpropagation

To visualize the features emphasized by the model, we applied Guided Backpropagation (GBP) to generate saliency maps. GBP aims to identify the features of the image that positively contribute to neuronal activation. It achieves this by combining two key mechanisms: (1) during backpropagation through ReLUs, only positive activations from the preceding layer are considered, and (2) during the backward pass, only positive error signals are propagated, as in DeconvNets. This process effectively disregards inhibitory influences (negative gradients), focusing exclusively on excitatory contributions. To obtain saliency maps for each input image, a variation of the original guided backpropagation algorithm was adapted to deal with 3D images (Springenberg et al., 2014). This adaptation generated a volumetric representation of feature activations by concatenating feature maps at each layer, thereby creating a 3D volume of the same size as the network input. The resulting saliency map highlights voxels assigned high positive values, which reflect regions that strongly influence the model's internal activations. These saliency maps can be overlaid on the corresponding 3D T1 image to visualize the anatomical locations receiving the strongest positive relevance signals from the model.

Despite its effectiveness for highlighting spatial details, the use of GBP as the primary attribution method can introduce inherent methodological limitations. Prior studies have shown that GBP can behave primarily as an edge detector or a partial image reconstruction mechanism, generating maps that emphasize local structural features rather than faithfully reflecting the representations learned by the model or its task-specific reasoning (Adebayo et al., 2018). In addition, visual saliency methods may exhibit inconsistencies and lack the robustness necessary for clinical interpretation, especially in critical domains such as medical imaging (Stubbin et al., 2024).

To address this limitation, GBP results were not interpreted in isolation. Instead, we implemented a dual-attribution approach by incorporating Integrated Gradients as a second, independent attribution method, and subsequently integrated both into a broader analytical framework combining voxel-wise statistical analysis and radiomic feature extraction within saliency-based VOIs. This approach reduces the subjectivity inherent to visual inspection and enables a more objective validation of the anatomical and microstructural relevance of the regions highlighted by the model, a strategy that has recently been promoted in the field of medical imaging (Brima and Atemkeng, 2024).

#### 2.4.2 Integrated gradients

Building upon this dual-attribution framework, Integrated Gradients (IG) (Sundararajan et al., 2017) was selected to mathematically counterbalance the heuristic nature of GBP. While GBP is highly sensitive to local structural edges, IG provides a robust, axiomatic attribution. It achieves this by computing the integral of gradients along a straight path from a baseline reference to the input image, ensuring that the resulting saliency maps satisfy the completeness axiom. In this work, we utilized a zero-intensity baseline (a black volume) and computed the attributions using a Riemann sum approximation with 50 interpolation steps. To mitigate the high-frequency noise and spatial discontinuity of gradients commonly observed in deep Vision Transformers and 3D Convolutional architectures (Smilkov et al., 2017), the IG algorithm was combined with a SmoothGrad approach. For each volume, we averaged the squared attributions across five iterations, injecting Gaussian noise with a standard deviation equal to 10% of the input volume's standard deviation. The interpretability algorithms were executed utilizing the Captum library (Kokhlikyan et al., 2020).

#### 2.4.3 Saliency maps preprocessing

Subsequently, the saliency maps generated by both GBP and IG were masked using the standard MNI152 brain mask, computed for each subject during the pre-processing procedure, to strictly exclude non-brain voxels. Because the two interpretability algorithms produce fundamentally different noise distributions, algorithm-specific baseline corrections were required to eliminate non-relevant, low-weight voxels prior to group-level comparisons.

For the GBP maps, which exhibited subject-specific baseline offsets, each map was mode-centered to zero. Conversely, for the IG maps, which inherently exhibited dense high-frequency noise despite SmoothGrad augmentation, a strict 90th-percentile threshold was applied to isolate only the highest-confidence activations. Following these method-specific baseline corrections, a universal intensity normalization step was applied to all maps to standardize the range of attention weights across the dataset, ensuring robust cross-subject comparability.

Once the saliency maps were standardized for each subject, spatial smoothing was applied using a 3D Gaussian kernel to further reduce residual noise and improve the signal-to-noise ratio of the attribution maps prior to group-level analysis. Following standard neuroimaging practice, an 8 × 8 × 8 mm full width at half maximum (FWHM) kernel was initially used, as commonly adopted in voxel-based morphometry (VBM) studies to account for residual inter-subject anatomical variability (Penny et al., 2011; Ashburner and Friston, 2000).

**Figure 3:**
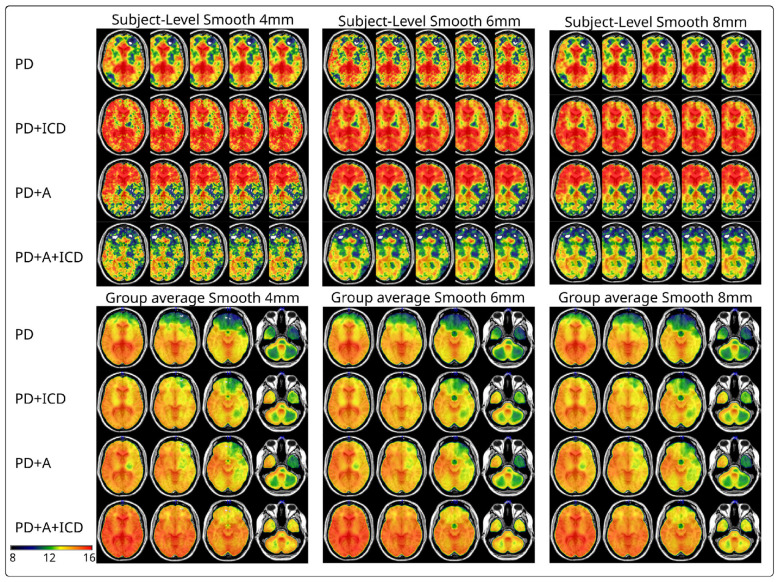
Sensitivity analysis of the smoothing parameter applied to Guided Backpropagation saliency maps. The upper block shows representative subject-level maps for each clinical subgroup, while the lower block shows the corresponding group-average maps. Columns correspond to Gaussian smoothing kernels of 4, 6, and 8 mm full width at half maximum (FWHM). Across smoothing configurations, the main spatial relevance patterns are broadly preserved, particularly in bilateral prefrontal, temporal, and cerebellar regions, suggesting that the observed anatomical trends are not strongly dependent on this preprocessing parameter.

To examine whether the main anatomical patterns were dependent on a single preprocessing setting, an additional sensitivity analysis was conducted by varying the smoothing kernel size. Specifically, smoothing was repeated using 4 and 6 mm Gaussian kernels, and the resulting subject-level and group-average saliency maps were visually compared across configurations. As illustrated in Figure 3, the principal spatial patterns of relevance, particularly in bilateral prefrontal, temporal, and cerebellar regions, were broadly preserved across smoothing levels. These observations suggest that the main explainability results are reasonably stable with respect to the choice of smoothing parameter.

#### 2.4.4 Image statistical analysis

The SPM12 toolbox was also used for the statistical procedure. Once the saliency map images were smoothed, the same procedure for VBM analysis was applied (Ashburner and Friston, 2000). Thus, for saliency maps, ANOVA was used to identify brain regions exhibiting significant differences in attribution intensity across the four clinical groups.

To account for morphological differences in head size, biological sex and Total Intracranial Volume (TIV) were incorporated as nuisance covariates in the model. This adjustment controls for global brain size variability, ensuring that observed differences in saliency intensity are not confounded by individual anatomical differences.

Finally, *post-hoc* pairwise comparisons were applied to determine the statistically significant group differences between PD vs PD + A, PD + ICD and PD + A + ICD. The Family-Wise Error (FWE) correction at *p* ≤ 0.05 was used for all cases corrected for multiple comparisons.

#### 2.4.5 Feature extraction

Although the previous analysis identified brain regions showing group-related differences in model-derived saliency patterns, they did not explicitly assess whether each experimental group exhibited distinct and characteristic neuroanatomical profiles.

**Figure 4:**
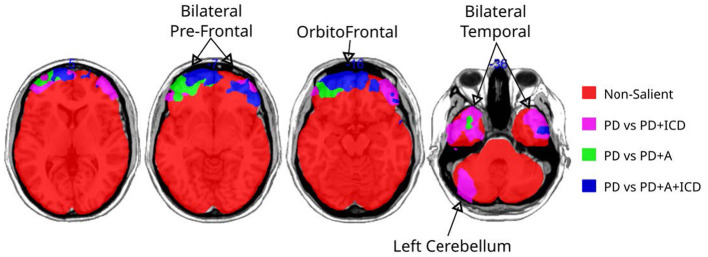
Binary VOIs created from the saliency maps depicted in Figure 5 to extract anatomical and texture-based features using CAT12 and PyRadiomics. The figure provides an anatomical summary of the main saliency-derived regions, highlighting bilateral prefrontal, orbitofrontal, temporal, and left cerebellar territories, with prominent patterns observed in the PD vs. PD + A + ICD comparison.

To address this, the statistical contrast maps were binarized and transformed into binary image masks as shown in Figure 4. The mask was then applied to the corresponding T1-weighted MRI volume of each participant, isolating thus the volumetric regions of interest (VOIs) associated with each saliency-based contrast. This procedure yielded subject-specific VOIs for each condition under investigation.

To provide a baseline of regions not highlighted by the explainability framework, we additionally analyzed non-salient regions, defined as the complement of the group-level saliency map relative to the whole brain volume. The inclusion of this broad, heterogeneous baseline serves as a negative control to verify that the statistical differences found in texture features are specific to the regions prioritized by the model's internal activations, rather than being a global property of the entire brain volume.

**Figure 5:**
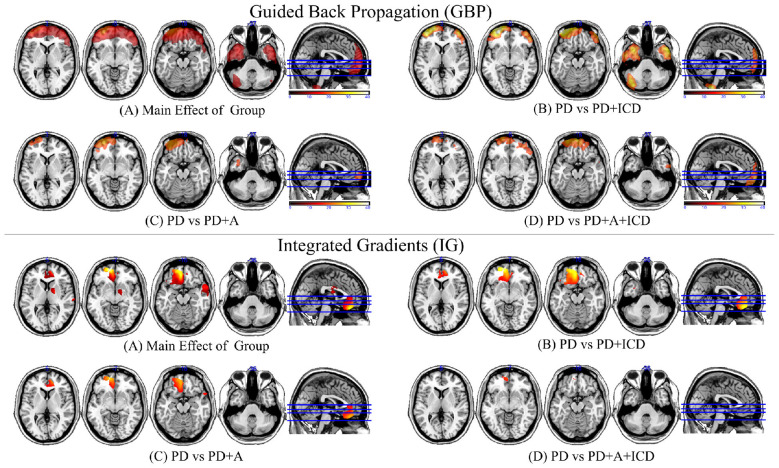
Between group differences of the saliency maps using ANOVA (biological sex and TIV of each participant was used as a nuisance variables). Top row depict the results from the GBP method, and the bottom row from the IG. In both cases, **(A)** depicts the main effect of group evaluated with an *F*-test; the next comparisons were evaluated with an *T*-test: **(B)** differences of the saliency maps between PD and PD + ICD; **(C)** PD vs. PD + A; and **(D)** PD vs. PD + A + ICD. No significant differences between the neuropsychiatric sub-groups were found. All contrasts were evaluated using *p* < 0.05, FWE corrected for multiple comparisons.

Subsequently, to elucidate the model's attentional mechanisms underlying the classification of each neuropsychiatric subgroup, we extracted quantitative features from each VOI per subject using two complementary approaches: (i) morphological and volumetric descriptors were derived using the Computational Anatomy Toolbox (CAT12) (Gaser et al., 2024), and (ii) radiomic texture features were computed via the PyRadiomics library (van Griethuysen et al., 2017).

##### 2.4.5.1 Morphological features

CAT12 (Gaser et al., 2024) under SPM12 and Matlab R2016b was used to extract the anatomical features of the corresponding VOIs. Starting from the pre-processed T1-weighted images, the corresponding averages of volumes, surfaces, sulcal depth, gyrification, cortical complexity (three fractal dimensions) and cortical thickness were then extracted for the VOI of each participant and each group (seven features for each VOI). We used the Schaefer 600 atlas (Schaefer et al., 2018) to obtain the features corresponding to the parcels located in each VOI for each subject. The resulting matrix was exported as a .csv file containing the average of the seven features calculated for each VOI, for each subject and group: PD, PD + A, PD + ICD and PD + A + ICD.

##### 2.4.5.2 Texture-based features

To ensure an independent anatomical validation and avoid circularity in our pipeline, Pyradiomics version 3.1.0 (Computational Imaging and Bioinformatics Lab, Harvard Medical School, Boston, MA, USA) was used to extract texture-based features (van Griethuysen et al., 2017). Instead of extracting features directly from the raw saliency masks, we utilized the standard anatomical parcels from the Schaefer 600 atlas that spatially overlapped with the salient regions identified by the model. This library allowed us to obtain 94 additional texture features for each of these anatomically independent, saliency-guided regions per subject. The resulting matrix was exported as a .csv file to perform further statistical analysis.

#### 2.4.6 Feature's statistical analysis

Subsequently, a statistical evaluation was performed for each extracted feature obtained by CAT12 and Pyradiomics. Given the non-normal distribution and unequal sample sizes across groups, the non-parametric Kruskal-Wallis test was applied to assess group-level differences for each feature (PD vs. PD + A, PD + ICD, and PD + A + ICD). This test was run for each feature obtained from each VOI depicted in Figure 4: 4 VOIs × 94 features = 376. Feature comparisons with a significance level of *p* ≤ 0.05 were retained for further analysis. Next, *post-hoc* comparisons for features that show significant differences were performed using the Dunn test (Dunn, 1964), a non-parametric pairwise comparison method. Bonferroni correction (Dunn, 1961) was applied to adjust p-values for multiple comparisons, reducing the risk of type I error. The Dunn test identified specific group differences, providing robust statistical support for feature selection.

Statistical analysis was implemented in Python using the Pingouin library (Vallat, 2018) for Kruskal-Wallis tests and Scikit (Terpilowski, 2019) for Dunn's *post-hoc* comparisons. This analysis provides a comprehensive framework for identifying significant differences between groups in the VOI's anatomical and textures-based features derived from the saliency maps. This ensures robust statistical validation of group differences and saliency maps.

## 3 Results

This section presents the explainability driven analyzes derived from the MobileViT-3D model. The first subsection examines voxel wise saliency patterns generated through Guided Backpropagation; evaluating the group related differences of the saliency maps. The second subsection reports the statistical evaluation of anatomical and radiomic features extracted from saliency derived regions of interest (VOIs), assessing whether these regions exhibit measurable structural or textural differences among the four clinical subgroups of PD. Together, these analyzes provide a multilevel characterization of the neuroanatomical patterns highlighted by the explainability framework.

### 3.1 Explainability analysis

Figure 5 presents the between-group differences in the saliency maps obtained by the Guided Backpropagation and Integrated Gradient algorithms. For both cases, Figure 5A shows the main effect of the group, identifying brain regions where the model's attribution intensities were significantly influenced across all groups. Figures 5B–D illustrate the *post-hoc* contrasts: (B) compares PD vs. PD + ICD, (C) contrasts PD vs. PD + A, and (D) examines PD vs. PD + A + ICD. Although consistent activation patterns were observed in the frontal and temporal regions, no statistically significant differences were identified between the neuropsychiatric subgroups using *p* < 0.05, corrected for multiple comparisons by Family Wise Error (FWE). The results are consistent with those previously found in work (Maggi et al., 2024) using the statistical Voxel-based morphometry technique, in which the neuropsychiatric PD groups had a reduced cortical density mainly in the prefrontal and orbitofrontal regions.

A visual sensitivity analysis was performed to evaluate the effect of the smoothing parameter on the saliency maps. Across the 4 mm, 6 mm, and 8 mm Gaussian kernels, the main relevance distributions remained qualitatively similar, with the most prominent patterns consistently observed in bilateral prefrontal, temporal, and cerebellar regions (Figure 3). Although minor variations in spatial extent were observed, no substantial changes in the general anatomical location of the saliency patterns were detected.

Due to the additional sensitivity analysis using the Integrated Gradients technique to obtain the saliency maps for each subject, the presence of stable and spatially coherent saliency patterns in both cases, despite the absence of voxel-wise group differences (between the neuropsychiatric subgroups) suggests that Guided Backpropagation captures common relevance profiles across subjects. Importantly, the saliency maps produced using Integrated Gradients displayed spatial distributions similar to those obtained with Guided Backpropagation, highlighting prefrontal, temporal, and orbitofrontal areas, though with a smaller extent than GBP. For GBP, the model consistently assigned the highest relevance to bilateral prefrontal, orbitofrontal, temporal, and left cerebellar regions, thereby demonstrating that the identified anatomical focus remains stable across both gradient-based approaches. This motivates the subsequent analysis of the GBP-derived regions of interest (VOIs), where anatomical and texture-based properties can be quantitatively evaluated to determine whether more subtle structural or radiomic distinctions emerge beyond the voxel-wise explainability maps.

### 3.2 Statistics from the VOI's features

The non-parametric statistical analysis of anatomical features obtained from CAT12 resulted in non-significant differences between the groups. Thus, we did not find significant differences in the morphology of the prefrontal regions in which the AI algorithm paid attention to classify the neuropsychiatric groups. We also found no significant differences for the non-salient regions. This means that no significant differences were found between the classes in the following features related to the morphological or anatomical shape of the whole brain, parceled according to the saliency maps obtained by the guided backpropagation algorithm: volumes of WM, GM and CSF, surfaces, gyrification, three surface complexity (estimated through fractal dimensions), sulcal depth, and cortical thickness.

Using the Pyradiomics library, which quantified 94 different features for each VOI depicted in Figure 4. The non-parametric statistical analysis performed for each feature and each VOI revealed no significant differences between groups for the features obtained from the non-salient region (red in Figure 4). This lack of significance in the non-salient baseline reinforces the validity of the explainability framework, suggesting that the model successfully ignored neuroanatomically irrelevant areas and concentrated on regions with truly discriminative textural information.

For the VOIs corresponding to the comparisons PD vs. PD + ICD, PD vs. PD + A, and PD vs. PD + A + ICD (violet, green, and blue in Figure 4), we found a significant main effect for different texture-based features using the Kruskal–Wallis test. Figure 4 also provides an anatomical summary of these saliency-derived regions, highlighting bilateral prefrontal, orbitofrontal, temporal, and left cerebellar areas. The *post-hoc* Dunn test performed subsequently revealed that some comparisons were not significant after Bonferroni correction for *p* < 0.05. Thus, for the features of the VOI derived from the PD vs. PD + A comparison, the test's significance did not pass the correction. Specifically, from the features corresponding to the VOI obtained from PD vs. PD + ICD, we found significant differences between PD + A and PD + ICD for *first-order kurtosis*. Interestingly, for the VOI corresponding to the PD vs. PD + A + ICD comparison, we found robust significant differences in eight different texture-based features. Notably, all eight of these features significantly differentiated the baseline PD group from the highly comorbid PD + A + ICD group, while specific short-run emphasis metrics also successfully differentiated the isolated PD + ICD group from the PD + A + ICD group. Despite that the main objective of this work is to present the explainability framework. It is important to note that the PD + A + ICD subgroup is considerably smaller than the other groups, which may affect the stability of the observed differences. Therefore, the apparent strength of the discrimination involving this subgroup should be interpreted with caution, as it may be influenced by sample size and class imbalance.

**Figure 6:**
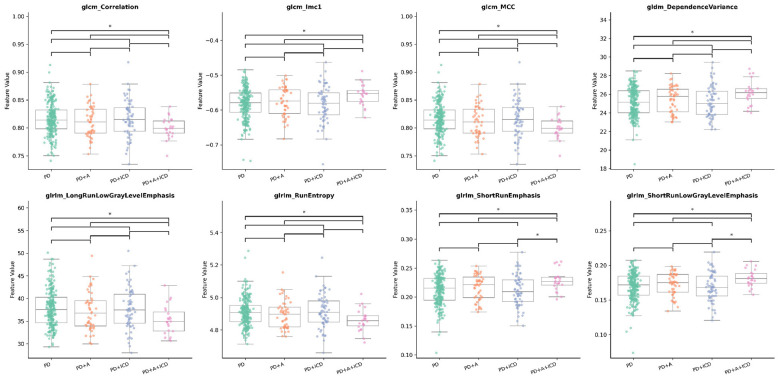
Data distributions of the significant radiomic features across all clinical cohorts. Boxplots overlaid with individual patient data points illustrate the dispersion, medians, and interquartile ranges for the eight texture-based features exhibiting significant main effects. All four clinical groups (PD, PD + A, PD + ICD, PD + A + ICD) are presented to provide full transparency of the cohort distributions. All eight panels display features extracted from the Blue VOI (the salient regions derived from the PD vs. PD + A + ICD baseline comparison). Statistical significance between specific subgroups, calculated via Dunn's *post-hoc* test with Bonferroni correction, is indicated by brackets (^*^p < 0.05). While significant pairwise median differences exist between the baseline and severe groups, the visual overlap in the raw data distributions underscores the subtle, high-dimensional nature of these microstructural alterations, reinforcing the necessity of deep learning architectures for robust patient classification.

To assess the biological and clinical relevance of these findings beyond statistical significance, we calculated the overall effect sizes (ϵ^2^) and extracted the medians with interquartile ranges (IQR). The magnitude, direction, and specific neuroanatomical meaning of these significant textural alterations are detailed comprehensively in Table 5. Furthermore, to explicitly visualize the practical separability, variance, and overlap of these microstructural features, the raw data distributions across all four clinical cohorts are presented as boxplots with overlaid individual data points in Figure 6, inclusive of statistical significance brackets for the *post-hoc* pairwise comparisons.

**Table 5:** Significant differences in Dunn's *post-hoc* test for texture-based features between PD and PD + A + ICD, including effect sizes and uncertainty measures.

Feature	Adj. *p*-val	Effect size (ϵ^2^)	PD median (IQR)	PD + A + ICD median (IQR)	Meaning
GLCM correlation	0.029	0.024	0.814 (0.034)	0.799 (0.022)	PD + A + ICD presents weaker linear dependency between neighboring pixel intensities compared to PD.
GLCM IMC1	0.032	0.024	–0.579 (0.055)	–0.553 (0.032)	PD + A + ICD shows increased texture complexity compared to PD.
GLCM MCC	0.029	0.024	0.814 (0.034)	0.799 (0.022)	PD + A + ICD shows increased texture complexity compared to PD.
GLDM dependence variance	0.029	0.022	25.145 (2.370)	26.183 (1.024)	PD + A + ICD presents increased variability in pixel intensities compared to PD.
GLRLM long run low gray level emphasis	0.026	0.025	37.607 (5.753)	34.944 (4.197)	PD + A + ICD presents decreased long homogeneous regions of dark pixels compared to PD.
GLRLM run entropy	0.025	0.023	4.906 (0.100)	4.857 (0.063)	PD + A + ICD presents decreased heterogeneity of texture patterns compared to PD.
GLRLM short run emphasis	0.016	0.024	0.215 (0.038)	0.227 (0.014)	PD + A + ICD presents increased fine textures compared to PD.
GLRLM short run low gray level emphasis	0.012	0.026	0.172 (0.027)	0.181 (0.014)	PD + A + ICD presents increased heterogeneity in dark areas compared to PD.

Adjusted *p*-values reflect Dunn's *post-hoc* comparisons between the baseline PD and PD + A + ICD groups. Epsilon-squared (ϵ^2^) denotes the overall Kruskal–Wallis effect size. Medians and interquartile ranges (IQR) illustrate the magnitude and variance of the focal texture differences.

An explanation of each computed feature is provided in the pyradiomics documentation and are defined as follows: GLCM correlation: is a value between 0 (uncorrelated) and 1 (perfectly correlated). It quantifies how a pixel's gray level is statistically related to its adjacent voxels; GLCM IMC1 quantifies the complexity of the texture using the mutual information matrix, with a higher value indicating a more complex tissue texture; GLCM MCC the maximal correlation coefficient, measures the complexity of the texture, with a lower value indicating a more complex texture; GLDM dependence variance measures how much pixel intensities deviate from the mean intensity, providing insight into the heterogeneity of the image in dependence on the size of the VOI.; long run low gray level emphasis higher values indicate extended regions of the same dark intensities; run entropy measures the uncertainty/randomness in the distribution of run lengths and gray levels. A higher value indicates more heterogeneity in the texture patterns; short run emphasis is a measure of the distribution of short run lengths, with a higher value indicative of shorter run lengths and finer textural textures; short run low gray level emphasis short runs indicate fine texture or heterogeneity in dark areas, emphasizing small isolated dark regions.

These results indicate that the Mobile-ViT model assigns higher relevance to bilateral prefrontal, orbitofrontal, temporal, and left cerebellar regions, capturing subtle intensity-derived texture properties that differentiate the neuropsychiatric subgroups. The findings suggest that these regions contain measurable variations in their textural signals, which the model highlights consistently in its saliency maps. Overall, the results show a clear correspondence between the areas emphasized by the model and the regions where radiomic features detect meaningful differences, providing a straightforward and coherent interpretation of the saliency maps.

## 4 Discussion

In this study, a fully 3D ViT-based AI architecture was employed to analyze structural T1-weighted MRI from idiopathic PD patients and PD patients with neuropsychiatric manifestations. In parallel, an explainability framework was developed to interpret the model's behavior through the integration of saliency maps and non-parametric statistical analyzes. Although we were unable to find any characteristic anatomical malformations that would identify each group, the model consistently assigned higher relevance to subtle texture variations in bilateral prefrontal, temporal, and left cerebellar regions, suggesting the presence of microstructural signal differences associated with the neuropsychiatric subtypes of PD.

Explainable Artificial Intelligence (XAI) plays a critical role in bridging the gap between black-box deep learning models and clinical interpretability, particularly in medical imaging, where trust and transparency are paramount. In the context of T1-weighted MRI analysis, XAI has enabled the identification of discriminative neuroanatomical patterns, offering visual insight into the model's decision-making process. Recent studies have shown that attention-based visualization techniques, such as Guided Backpropagation, Grad-CAM, and integrated gradients, can successfully highlight brain regions associated with disease phenotypes without explicit anatomical labeling (Luna et al., 2022). The 3D Vision Transformer architecture consistently assigned high relevance to prefrontal, temporal, and cerebellar territories, aligning with known circuits implicated in PD-related behavioral symptoms. Unlike tumor detection studies, where heterogeneous mass boundaries provide high-contrast features for learning (Zeineldin et al., 2022; Windisch et al., 2020), the neuropsychiatric subgroups examined here exhibit largely normal-appearing morphometry. This required the integration of *post-hoc* statistical validation and feature-based analysis to confirm that the saliency patterns corresponded to systematic texture-level differences rather than noise. Together, these findings suggest that AI explainability methods are useful not only for localization but also for uncovering texture and intensity-based cues that may underlie early neurodegenerative changes. In this line, recent studies reveal distinct brain abnormalities related to changes in brain texture in subcortical regions in patients with refractory trigeminal neuralgia (Danyluk et al., 2021). Patients with Amyotrophic Lateral Sclerosis showed alterations in small clusters of subcortical gray and white matter associated with disease progression (Parnianpour et al., 2024), and patients with Alzheimer's disease showed texture alterations of T1-weighted MRI associated with regional tau burden in the allocortical and pericortical regions (Lee et al., 2021). Additionally, several studies have applied radiomics methodologies to extract a large number of quantitative features from previously predefined regions of interest (ROIs) in the brain using T1-weighted MRI. For example, the work by Bu et al. combines conventional T1-weighted imaging with susceptibility-weighted imaging (SWI) and extracts thousands of texture-based radiomics features from subcortical nuclei to differentiate idiopathic PD from Multiple System Atrophy (MSA) (Bu et al., 2023). Similarly, Panahi and Hosseini (2024) developed a multi-modality radiomics approach that integrates features from both T1-weighted imaging and diffusion tensor imaging (DTI) to differentiate PD motor subtypes such as tremor-dominant (TD) vs. postural instability gait difficulty (PIGD) using machine learning classifiers; anatomical structures such as the thalamus, substantia nigra, and putamen play a key role in this differentiation. In addition, Tupe-Waghmare et al. (2021) showed that radiomic descriptors extracted from routine T1-weighted MRI capture microstructural variability in regions such as the nucleus accumbens and the ventral diencephalon, demonstrating that texture-based features can sensitively encode disease-related heterogeneity even in morphologically normal-appearing tissue.

Our findings are consistent with previous studies (Maggi et al., 2024; Reijnders et al., 2010), which reported that neuropsychiatric manifestations in Parkinson's disease predominantly affect the frontal cortex. However, those studies employed Voxel-Based Morphometry and demonstrated reduced cortical density in PD patients with neuropsychiatric symptoms. Our analysis did not reveal significant differences in conventional anatomical features such as cortical thickness, gyrification, or cortical complexity. Instead, we identified alterations in cortical texture that may reflect microstructural changes detectable as voxel intensity variations, thus offering compatibility with VBM findings. Specifically, our rigorous *post-hoc* statistical analysis revealed that the most prominent texture alterations were driven by the contrast between the baseline PD group and the highly comorbid PD + A + ICD group within the salient VOIs. Furthermore, specific textural metrics, particularly those related to *short-run emphasis*, were also able to significantly differentiate patients with isolated impulse control disorders (PD + ICD) from those with combined comorbidities (PD + A + ICD). Interestingly, the isolated apathy group (PD + A) did not exhibit statistically robust pairwise textural differences from the baseline or severe groups after strict Bonferroni correction. As explicitly visualized in the raw data distributions (Figure 6), these individual texture features exhibit substantial numerical overlap across the clinical spectrum. This high degree of overlap highlights exactly why traditional univariate thresholding struggles to separate these cohorts, and strongly reinforces the necessity of using the high-dimensional pattern recognition capabilities of a 3D Vision Transformer to capture the complex, multifocal signatures of neuropsychiatric PD phenotypes.

According to the sensitivity test using two different methodologies to extract the saliency maps, both GBP and IG converged on the orbitofrontal cortex (OFC) as a key region distinguishing PD neuropsychiatric subgroups, consistent with prior structural MRI literature reporting reduced cortical thickness in the OFC and vmPFC across PD-ICD and PD-apathy populations (Gu et al., 2022; Cordeiro et al., 2025). GBP additionally captured differences in prefrontal, temporal, and cerebellar regions, aligning with findings of gray matter reductions in frontal, temporal, and cerebellar areas associated with the co-occurrence of apathy and ICD in PD (Maggi et al., 2024). In contrast, IG yielded a more spatially focused pattern with a left-hemisphere tendency, which may reflect its inherent methodological properties: by averaging gradients along an interpolation path from a baseline, IG produces attribution maps that are less sensitive to local gradient fluctuations, potentially prioritizing only the most robust structural discriminators (Sundararajan et al., 2017). GBP, while capturing a broader set of discriminative regions, is known to propagate only non-negative gradients and can be more susceptible to noise, potentially explaining its wider spatial extent. Together, the overlap in orbitofrontal regions across both methods reinforces the central role of OFC structural integrity in PD neuropsychiatric heterogeneity, while the discrepancies highlight the importance of using complementary explainability approaches when interpreting deep learning models applied to structural neuroimaging data.

The 8 mm FWHM smoothing kernel was initially selected following standard VBM practice to account for inter-subject anatomical variability. To assess whether the main explainability findings were dependent on this preprocessing choice, an additional sensitivity analysis was conducted using 4 mm and 6 mm Gaussian smoothing kernels. Across configurations, the principal spatial patterns of relevance were broadly preserved, particularly in bilateral prefrontal, temporal, and cerebellar regions, suggesting that the main anatomical conclusions are not strongly dependent on the choice of smoothing parameter. Nevertheless, it is important to note that the robustness analysis was limited to preprocessing variability, specifically the choice of smoothing parameter. By incorporating both Guided Backpropagation and Integrated Gradients, our framework ensures that the observed saliency patterns are robust across different attribution mechanics. The spatial consistency between both techniques strongly suggests that the model is genuinely relying on these prefrontal, temporal, and cerebellar features for its predictions, rather than the patterns being artifacts of a single algorithm. In relation to the smoothing analysis, variations in kernel size slightly affected the spatial extent of the resulting VOIs; however, the core brain territories highlighted by the model remained stable. Additionally, the non-salient comparator region is spatially heterogeneous, which limits the strength of inference that can be drawn from it as a negative control. The lack of significant texture differences in this region supports the interpretation that the observed microstructural alterations are more specifically associated with the neuroanatomical areas prioritized by the model, rather than reflecting global brain texture effects.

Although previous studies have applied artificial intelligence methods to analyze Parkinson's disease and other neurological disorders, these approaches typically rely on predefined anatomical hypotheses or focus primarily on improving predictive accuracy. In contrast, the proposed framework uses saliency maps derived through Guided Backpropagation to identify data-driven regions of interest without imposing prior anatomical constraints. The integration of radiomics-based feature extraction and non-parametric statistical testing enables the characterization of latent neuroanatomical patterns underlying the model's relevance signals. This multimodal analytical approach provides a principled pathway interpreting the internal representations of deep learning models and for uncovering subtle microstructural variability that may contribute to the heterogeneity of neuropsychiatric manifestations in Parkinson's disease.

The proposed workflow, based on T1 imaging analysis using XAI, could potentially serve as a foundational tool for multidisciplinary team meetings, where such interpretable outputs might eventually complement standard clinical assessments and support future research aimed at better understanding the neuropsychiatric effects of Parkinson's disease on the brain. While these preliminary findings are promising, further prospective studies are necessary to evaluate the framework's contribution to the identification, consistency, and clinical interpretability of disease-relevant brain regions across experts. Such steps will be essential for exploring the long-term research and translational value of these methods.

Given the between-group imbalance in sample size and the exploratory nature of the study, these results should be interpreted as hypothesis, rather than definitive evidence of neuropsychiatric disorder effects on brain morphology in the Parkinson's disease population. This work presents a possible morphological change which may occur in patients' brains and could potentially be observable on MRI images. Nevertheless, the observed patterns offer valuable direction for future research by highlighting potential alterations in brain morphology that may be influenced either by the disease itself or by other unknown factors.

A methodological limitation of the current study is related to imaging acquisition variability. Although our preprocessing pipeline helps mitigate site-related variability, our statistical models did not explicitly account for scanner manufacturer or inter-protocol variability. Given the subtle nature of the texture differences reported, residual batch effects cannot be fully excluded. Future work should therefore validate these radiomic descriptors in an independent, larger, and more balanced cohort, incorporating site harmonization methods such as ComBat, to provide unbiased confirmation of the reported texture differences and assess whether these patterns remain stable across multicenter populations. Furthermore, while extracting radiomic features from standard anatomical parcels substantially mitigates circularity bias, a residual dependency remains because the selection of these specific parcels was inherently guided by the model's initial saliency maps. Consequently, the current framework is best suited for generating data-driven hypotheses, and fully independent confirmation using pre-specified regions of interest will be required in future studies.

While our framework identified eight significant radiomic features, the imbalance in sample size, particularly the small PD + A + ICD group (*n* = 23), remains a critical limitation. This disparity suggests that the reported texture-based findings should be viewed as exploratory hypotheses rather than definitive evidence. Specifically, the stability and generalizability of these eight features may be sensitive to the limited representation of the comorbid phenotype, necessitating further validation in larger, more balanced cohorts to ensure these microstructural signatures are truly robust across diverse Parkinson's populations.

Future work will also focus on strengthening these findings in two directions. First, beyond single-protocol T1-weighted imaging, future studies could evaluate whether the proposed XAI framework can be extended or complemented with additional neuroimaging modalities, when available, to provide a more comprehensive characterization of disease-related brain alterations. Such multimodal investigations may help determine whether the anatomical patterns identified here remain consistent across complementary imaging sources. Second, future studies should continue to expand this explainability component by evaluating transformer-specific attention mapping mechanisms to further validate the stability of the anatomical signatures across subjects and subgroups. While the current framework provides a novel pathway for characterizing PD phenotypes, the reliance on a relatively small set for the PD + A + ICD group remains a constraint on the generalizability of the texture-based results. Future validation should therefore test these radiomic descriptors in larger, more balanced and independent cohorts, ideally using externally defined anatomical ROIs and complementary attribution methods to assess whether the reported patterns remain stable under a more confirmatory study design.

## 5 Conclusion

This work presents a fully 3D Vision Transformer-based framework for analyzing structural MRI in Parkinson's disease and its neuropsychiatric subtypes, using a cohort of 364 T1-weighted scans from the PPMI database. By integrating MobileViT-3D with two complementary *post-hoc* explainability techniques (Guided Backpropagation and Integrated Gradients), the approach robustly highlights the neuroanatomical patterns in bilateral prefrontal, temporal, and cerebellar regions that may contribute to the model's decisions, offering an interpretable computational perspective for subgroup differentiation.

Although *post-hoc* analysis of these saliency maps showed no statistically significant differences between neuropsychiatric subgroups after FWE correction, conventional CAT12-derived morphometric features also showed no significant group differences. The subsequent radiomic analysis provided deeper insight. After applying Bonferroni correction to account for multiple comparisons, eight radiomic texture features remained significant with an adjusted *p* = 0.012–0.032, including texture descriptors associated with differences between the PD + A + ICD group from idiopathic PD. These findings suggest that combining explainability outputs with *post-hoc* statistical validation may help characterize subtle imaging patterns in morphologically normal-appearing tissue.

Overall, this study provides preliminary evidence that combining explainable deep learning with quantitative feature analysis may help uncover imaging patterns that are not readily captured by conventional approaches. This integrative strategy represents an exploratory step toward refining the characterization of neuropsychiatric phenotypes in Parkinson's disease and may serve as a basis for future research into clinically interpretable, personalized assessment tools.

## Data Availability

Publicly available datasets were analyzed in this study. This data can be found at: https://www.ppmi-info.org/data.
